# *NAT1* and *NAT2* genetic polymorphisms and environmental exposure as risk factors for oesophageal squamous cell carcinoma: a case-control study

**DOI:** 10.1186/s12885-015-1105-4

**Published:** 2015-03-18

**Authors:** Marco Matejcic, Matjaz Vogelsang, Yabing Wang, Iqbal M Parker

**Affiliations:** 1 International Centre for Genetic Engineering and Biotechnology, Cape Town Component, Observatory, UCT Medical Campus, Anzio Road, Observatory 7925, Cape Town, South Africa; 2 Division of Medical Biochemistry and IDM, UCT Faculty of Health Sciences, Cape Town, South Africa

**Keywords:** N-acetyltransferases, Single nucleotide polymorphism, Acetylator phenotype, Environmental carcinogens, Oesophageal cancer

## Abstract

**Background:**

Tobacco smoking and red meat consumption are some of the known risk factors associated with the development of oesophageal cancer. N-acetytransferases (*NAT1* and *NAT2*) play a key role in metabolism of carcinogenic arylamines present in tobacco smoke and overcooked red meat. We hypothesized that *NAT1* and *NAT2* genetic polymorphisms may influence the risk of oesophageal cancer upon exposure to environmental carcinogens.

**Methods:**

Single nucleotide polymorphisms (SNPs) in the *NAT1* and *NAT2* genes were investigated by genotyping 732 cases and 768 healthy individuals from two South African populations to deduce the acetylator phenotype (slow, intermediate or rapid) from the combination of the genotyped SNPs.

**Results:**

The 341 CC genotype (rs1801280) was significantly associated with a reduced risk for oesophageal cancer in the Mixed Ancestry population (OR = 0.31; 95% CI 0.11-0.87). The *NAT2* slow/intermediate acetylator status significantly increased the risk among cigarette smokers in the Black population (OR = 2.76; 95% CI 1.69-4.52), as well as among alcohol drinkers in the Mixed Ancestry population (OR = 2.77; 95% CI 1.38-5.58). Similarly, the *NAT1* slow/intermediate acetylator status was a risk factor for tobacco smokers in the Black population (OR = 3.41; 95% CI 1.95-5.96) and for alcohol drinkers in the Mixed Ancestry population (OR = 3.41; 95% CI 1.70-6.81). In a case-only analysis, frequent red meat consumption was associated with a significantly increased cancer risk for *NAT2* slow/intermediate acetylators in the Mixed Ancestry population (OR = 3.55; 95% CI 1.29-9.82; *P* = 0.019), whereas daily white meat intake was associated with an increased risk among *NAT1* slow/intermediate acetylators in the Black population (OR = 1.82; 95% CI 1.09-3.04; *P* = 0.023).

**Conclusions:**

Our findings indicate that N-acetylation polymorphisms may modify the association between environmental risk factors and oesophageal cancer risk and that N-acetyltransferases may play a key role in detoxification of carcinogens. Prevention strategies in lifestyle and dietary habits may reduce the incidence of oesophageal cancer in high-risk populations.

**Electronic supplementary material:**

The online version of this article (doi:10.1186/s12885-015-1105-4) contains supplementary material, which is available to authorized users.

## Background

Oesophageal squamous cell carcinoma (OSCC) is the most common histological subtype of oesophageal cancer in developing countries and the main cause of cancer death in Southern Africa, with incidence and mortality rates approximately 7-fold higher among men and 4-fold higher among women than those in Northern Africa [1]. The prevalence of oesophageal cancer varies widely between different regions of South Africa, with exceptionally high-incidence rates recorded in the Black African populations of KwaZulu-Natal and the Eastern Cape [2]. The wide geographical variation in incidence of OSCC is most likely due to both genetic and environmental factors [3]. A number of epidemiological studies have pointed to tobacco smoking and alcohol consumption as major environmental risk factors for OSCC [4-6], and recent studies have also reported a significant association between red meat intake and an increased risk of oesophageal cancer [7,8].

Heterocyclic amines (HCAs) and nitrosamines are among the major arylamine carcinogens present in tobacco smoke and overcooked meat [9,10]. In humans these compounds are metabolized by the Phase I and Phase II xenobiotic metabolizing enzymes. The N-acetyltransferases (NATs) are the main phase II xenobiotic metabolizing enzymes involved in either detoxification or activation of arylamines, depending on the nature of the substrate and the organ involved [11]. The human NATs are encoded by two genes, *NAT1* [HGNC:7645; Ensembl:ENSG00000171428] and *NAT2* [HGNC:7646; Ensembl:ENSG00000156006], separated by 170 kb on chromosome 8p21.3 − 23.1 [12].

Single nucleotide polymorphisms (SNPs) in the *NAT1* and *NAT2* genes alter the enzyme activity and are classified into rapid, intermediate or slow acetylators [13]. The relationship between *NAT2* polymorphisms and *NAT2* acetylation activity has been widely investigated and a strong genotype-phenotype correlation exists [14]. The common SNPs in the coding region of the *NAT2* gene, 191G>A, 341T>C, 590G>A, and 857G>A (referred to as NAT2*14, NAT2*5, NAT2*6, and NAT2*7 alleles respectively) lead to a significant decrease in acetylation activity compared to the wild-type allele (NAT2*4), and are thus designated as slow acetylator alleles [15]. A recent study reported that genotyping of these SNPs is sufficient to predict the *NAT2* acetylator status with high accuracy [16]. The NAT2*5, NAT2*6, and NAT2*7, and NAT2*14 alleles were observed at frequencies of 20%, 15%, 1.7%, and 2.5% in the South African Black population, respectively [17]. The relationship between *NAT1* polymorphisms and NAT1 acetylation activity, on the other hand, has been poorly investigated with inconsistent genotype-phenotype correlation for SNPs that lie outside the coding region [18]. The *NAT1* polymorphisms in Black South Africans are solely represented by NAT1*4 (wild-type), NAT1*10, and NAT1*3 alleles, with frequencies of 49%, 50%, and 1%, respectively [19]. The NAT1*10 allele, inferred by the simultaneous presence of two SNPs (1088T> A and 1095C>A) within the 3’-UTR of the *NAT1* gene, leads to an alteration of the consensus polyadenylation signal and is associated with a significantly increased acetylation activity compared to the wild-type allele [20]. The NAT1*3 allele is inferred by the sole presence of 1095C>A, and does not result in a significantly elevated acetylation activity compared to the wild-type allele [18].

*The NAT1* and *NAT2* polymorphic variants may influence the metabolism of arylamine carcinogens and modulate the individual susceptibility to cancer upon exposure to environmental risk factors [21]. Genetic polymorphisms in the *NAT1* and *NAT2* loci have been associated with susceptibility to bladder, colon and rectal, breast, head and neck, lung and prostate cancers (reviewed in [22]). However, more consistent results were observed when the polymorphisms were investigated in association with environmental exposure [23]. Previous studies have reported conflicting results with regard to the role of *NAT1* and *NAT2* polymorphisms in oesophageal carcinogenesis [24-29].

We believe that genetic polymorphisms associated with low N-acetylation activity may lead to an impaired deactivation of arylamine carcinogens making subjects with slow or intermediate acetylator status more susceptible to environmental or dietary carcinogens than those with the rapid acetylator status. This study investigated the role of *NAT1* and *NAT2* functional polymorphisms as independent risk factors for OSCC and as modifiers of the association between environmental risk factors and OSCC in two indigenous populations of South Africa.

## Methods

### Study group

The study group included 1500 South African subjects subdivided into 463 OSCC patients and 480 control individuals from the Black population, and 269 OSCC patients and 288 control individuals from the Mixed Ancestry population. The Black individuals were mostly Xhosa speakers residing in the Western Cape Province of South Africa. The Mixed Ancestry individuals in the Western Cape is an admixed population that originated about 300 years ago from the union of different ethnic groups, receiving ancestral contribution from the indigenous Khoisan, sub-Saharan Africans, Europeans, Indonesians and Malaysians [30].

All patients selected for the study were histologically diagnosed with squamous cell carcinoma of the oesophagus, recruited between 2000 and 2012 from Groote Schuur and Tygerberg Hospitals in Cape Town. The control group included healthy volunteers with no history of any cancer and no familial history of oesophageal cancer. They were frequency matched to cases for geographical location, ethnicity, sex, and age. Because oesophageal cancer is a disease of middle-age or the elderly, the minimum age of control individuals set for the study was 50. However, the control group had a higher proportion of individuals below the age of 60, while the patients were mostly over the age of 60. As a result, the mean age of the patients was approximately three years higher than that in control individuals in both the Black and Mixed Ancestry population groups (Table 1).

**Table 1:** **Characteristics of the population study and effect of tobacco smoking, alcohol consumption, and red meat intake on OSCC risk**

		Black				Mixed Ancestry			
Demographic/risk factors	Variables	Controls (%)	Cases (%)	OR (95% CI)^a^	*P*-value	Controls (%)	Cases (%)	OR (95% CI)^a^	*P*-value
Gender	Males	235 (49)	229 (49)			178 (62)	177 (66)		
	Females	245 (51)	234 (51)			110 (38)	92 (34)		
Age	Mean (SD^b^)	56.7 (15.0)	59.6 (10.7)			57.7 (14.3)	60.7 (10.3)		
Tobacco smoking	Never smokers	258 (54)	181 (39)	1 (Ref)		62 (22)	15 (6)	1 (Ref)	
	Smokers^c^	222 (46)	280 (60)	1.79 (1.39-2.33)	<0.0001	226 (78)	250 (93)	4.57 (2.53-8.27)	<0.0001
	Unknown	0 (0)	2 (0.4)			0 (0)	4 (1)		
Total cigarettes/day	0	258 (54)	181 (39)	1 (Ref)		62 (21)	15 (6)	1 (Ref)	
	<10	137 (28)	179 (39)	1.86 (1.39-2.49)	<0.0001	118 (41)	85 (31)	2.98 (1.59-5.59)	0.001
	≥10	73 (15)	95 (20)	1.85 (1.29-2.66)	0.001	90 (31)	160 (59)	7.35 (3.95-13.6)	<0.0001
	Unknown	12 (2)	8 (2)			18 (6)	9 (3)		
Alcohol drinking	Non-drinkers	201 (42)	175 (38)	1 (Ref)		115 (40)	51 (19)	1 (Ref)	
	Drinkers^d^	278 (58)	286 (62)	1.18 (0.91-1.53)	0.211	172 (60)	215 (80)	2.82 (1.92-4.15)	<0.0001
	Unknown	1 (0.2)	2 (0.4)			1 (0.3)	3 (1)		
Tobacco smoking + alcohol drinking	Never smokers^e^	257 (53)	180 (39)	1 (Ref)		62 (21)	15 (6)	1 (Ref)	
	Smokers/non-drinkers	41 (8)	33 (7)	1.15 (0.70-1.89)	0.583	75 (26)	37 (14)	2.04 (1.02-4.06)	0.042
	Smokers/drinkers	181 (38)	247 (53)	1.95 (1.49-2.55)	<0.0001	150 (52)	213 (79)	5.87 (3.22-10.7)	<0.0001
	Unknown	1 (0.2)	3 (0.6)			1 (0.3)	4 (1)		
Red meat intake	3 times/week or less	−	261 (56)			−	108 (40)		
	Daily or almost daily	−	37 (8)			−	50 (19)		
	Unknown	−	165 (36)			−	111 (41)		
White meat intake	3 times/week or less	−	151 (33)			−	75 (28)		
	Daily or almost daily	−	148 (32)			−	82 (30)		
	Unknown	−	164 (35)			−	112 (42)		
Fish	3 times/week or less	−	289 (62)			−	146 (54)		
	Daily or almost daily	−	9 (2)			−	11 (4)		
	Unknown	−	165 (36)			−	112 (42)		
Vegetables	3 times/week or less	−	243 (52)			−	54 (20)		
	Daily or almost daily	−	53 (11)			−	101 (37)		
	Unknown	−	165 (36)			−	114 (42)		
Fried food	3 times/week or less	−	291 (63)			−	135 (50)		
	Daily or almost daily	−	5 (1)			−	20 (7)		
	Unknown	−	167 (36)			−	114 (42)		

^a^Crude odds ratio was calculated.

^b^SD = standard deviation of the mean.

^c^Smokers = current and former smokers.

^d^Drinkers = light to heavy alcohol drinkers.

^e^Never smokers (including drinkers and non-drinkers).

Ref = reference allele.

Ethics approval was obtained from the joint University of Cape Town/Groote Schuur Hospital Research Ethics Committee and the University of Stellenbosch/Tygerberg Hospital Ethics Committee. Written informed consent was obtained from all study participants.

### Definition of variables

The study participants completed a standard questionnaire to collect demographic (i.e. ethnicity, origin, language, age, gender), lifestyle (i.e. smoking history, alcohol consumption), and dietary (i.e. frequency and type of meat intake) information.

The smoking habits were reported as smoking of cigarettes, hand-rolled cigarettes, and pipes (all of which referred to as ‘cigarettes’ in the text). Smoking is a qualitative variable and subjects were classified as current smokers (currently smoking at recruitment or stopped smoking not earlier than one year prior to recruitment), former smokers (smoked regularly at some point in their lives for at least six months or quitted smoking at least one year prior to recruitment), or never smokers (never smoked). Current and former smokers were also asked to report the number of cigarettes smoked per day and subjects were grouped into those who smoked <10 cigarettes/day and those who smoked ≥10 cigarettes/day.

Alcohol consumption status was defined according to the frequency of alcohol (wine or beer) consumed per week and subjects were subdivided into those who consumed more than one litre of wine/beer per week and those who consumed less than one litre of wine/beer per week.

The study participants were also asked to report their average intake of red and white meat, fish, vegetables, and fried food and grouped into those who consumed each food category less than three times per week and those who consumed them (almost) daily. Red meat included beef, veal, pork, lamb, and offal. White meat included chicken, turkey, and duck. Data on food consumption was not available for control individuals, and only 299 Black patients and 158 Mixed Ancestry patients (out of 463 and 269 total patients, respectively) provided information about meat, fish, vegetables, and fried food intake.

### Isolation and purification of DNA

Peripheral blood samples were collected from each subject enrolled in the study and stored at −20°C prior to DNA extraction. Genomic DNA was extracted using a standard protocol [31]. All DNA samples were diluted to a final concentration of 20 ng/μl in 96-well plates and stored at −20°C until genotyping.

### SNP selection and genotyping

Genotyping was determined blinded as to case-control status and environmental exposure of study participants.

Four *NAT2* SNPs; 191G>A (rs1801279), 341T>C (rs1801280), 590G>A (rs1799930), and 857G>A (rs1799931), were genotyped by TaqMan allele discrimination assay. Each reaction was carried out in a 2.5 μl volume containing 1X Universal PCR Master Mix, 1X Drug Metabolism Genotyping Assay Mix (Applied Biosystems), and 20 ng of DNA. The thermal cycling conditions consisted of an initial denaturation step at 95°C for 10 min, followed by 50 cycles of denaturation at 92°C for 15 sec and annealing/extension at 60°C for 90 sec. The amplification reaction and fluorescence measurement was performed in a Roche LightCycler 480 II, and genotypes were assigned using SP4 1.5.0 software (Roche). Five *NAT2* alleles were identified: NAT2*4 (wild-type), NAT2*5, NAT2*6, NAT2*7 and NAT2*14 (slow acetylator alleles). The acetylator phenotype was determined as follows: (i) subjects with two NAT2*4 alleles were classified as “rapid” acetylators; (ii) subjects with any combination of two slow acetylator alleles were “slow” acetylators; (iii) subjects with one wild-type allele and one slow acetylator allele were “intermediate” acetylators. Individuals carrying alleles deducing either slow or intermediate acetylator status were merged into one group, referred to as *NAT2* “slow/intermediate” acetylators, and compared to rapid acetylators (NAT2*4/*4).

Two *NAT1* SNPs; 1095C>A (rs15561) and 1088T>A (rs1057126), were determined by TaqMan assay as previously described [32]. We used a three-probe system to genotype the two variants simultaneously in a single reaction. This was necessary because the 1088/1095 SNPs are too close to each other to use the conventional two-probe TaqMan assay. Primers and hybridization probes were designed according to the recommendations by Roche Diagnostics Inc. [see Additional file 1]. 1X Universal PCR Master Mix (Applied Biosystems) was used as PCR solution and primers, probes, and DNA were added to final concentrations of 300 nM, 100 nM, and 8 ng/μl, respectively. The amplification reaction was carried out in a Roche LightCycler 480 II instrument with an initial hold step at 95°C for 10 min followed by 40 cycles of a two-step reaction (92°C for 15 s, 60°C for 1 min). Three *NAT1* alleles were identified: NAT1*4 (wild-type), NAT1*3 and NAT1*10 (rapid acetylator allele). Individuals with both 1095C>A and 1088T>A were referred to as NAT1*10 carriers, and those with only 1095C>A were NAT1*3 carriers. Subjects were classified as slow, intermediate, or rapid acetylators if they had zero, one, or two copies of the NAT1*10 allele, respectively. Individuals with either zero or one copy of NAT1*10 allele were merged into one group, referred to as *NAT1* “slow/intermediate” acetylators, and compared to rapid acetylators (NAT1*10/*10).

### Statistical analysis

Genotype counts among controls were tested for deviation from Hardy-Weinberg Equilibrium using the Chi-Square test with one degree of freedom [33].

The genetic association analyses were performed assuming a multiplicative genetic model for any risk allele or risk category. Where sample sizes were sufficiently large, we tested for differences in frequency distribution between cases and control individuals using Pearson’s Chi-Square (χ^2^) test of association. Crude odds ratio (OR), adjusted odds ratio, 95% confidence interval (95% CI), and *P*-value for association were estimated for the tested allele or category. The adjustment of the OR for potential confounders such as age, gender, tobacco smoking, and alcohol consumption was computed by using multivariate conditional logistic analysis [the disease status (case/control) was checked against each polymorphism (GG/GA/AA) for all the cases and controls used in the study by considering also other variables such as smoking, alcohol consumption, age and sex]. All reported *P*-values are two-sided and a *P*-value <0.05 was considered as significant. A conservative *P*-value threshold of 0.0042 (=0.05/[2x6]) was used to assess the significance of association applying Bonferroni correction for multiple testing of the six genotyped SNPs in the two populations.

Case-only analyses were performed to evaluate the interaction between the acetylation polymorphism and meat consumption by testing for frequency of red and white meat intake (<3 times per week/almost daily).

All statistical analyses were performed using SPSS 19 software package (SPSS, Chicago, IL). Linkage disequilibrium and haplotype analyses were performed on the control groups using Haploview [34]. The pairwise linkage disequilibrium was measured by variables D^I^ and r^2^. Pearson’s χ^2^ was used to test for differences in haplotype distribution between patients and control individuals using UNPHASED [35].

## Results

### Demographic details and environmental exposure

The distribution of demographic details and lifestyle factors among cases and control individuals enrolled in the study is shown in Table 1. The percentages of tobacco smokers, alcohol drinkers, and of subjects that consumed red meat, fried food, and vegetables daily were higher in the Mixed Ancestry group than in the Black group, while no significant difference was observed for daily consumption of white meat and fish.

Tobacco smoking was associated with a significantly increased risk of OSCC among both the Black (OR = 1.79; 95% CI 1.39-2.33; *P* < 0.0001) and Mixed Ancestry (OR = 4.57; 95% CI 2.53-8.27; *P* <0.0001) individuals. In the Mixed Ancestry group, the risk associated with tobacco smoking increased with the number of cigarettes smoked per day, with a 2.98-fold higher risk (95% CI 1.59-5.59; *P* = 0.001) among those smoking <10 cigarettes/day, and a 7.35-fold higher risk (95% CI 3.95-13.66; *P* < 0.0001) among those smoking ≥10 cigarettes/day, compared to never smokers. No cumulative effect of tobacco smoking on OSCC risk was observed in the Black group.

A higher proportion of alcohol drinkers was observed among patients compared to control individuals in both ethnic groups, although the difference was significant only in the Mixed Ancestry group (OR = 2.82; 95% CI 1.92-4.15; *P* < 0.0001). The analysis of the combined effect of tobacco smoking and alcohol consumption showed a significantly higher OSCC risk for smokers who drank alcohol (‘smokers/drinkers’) compared to ‘never smokers’ (including drinkers and non-drinkers) among both Black (OR = 1.95; 95% CI 1.49-2.55; *P* < 0.0001) and Mixed Ancestry (OR = 5.87; 95% CI 3.22-10.71; *P* <0.0001) individuals. No increased risk was observed for ‘smokers/non-drinkers’ in the Black group (*P* = 0.583), while only a weak association was detected in the Mixed Ancestry group (OR = 2.04; 95% CI 1.02-4.06; *P* = 0.042).

### Linkage disequilibrium analysis

**Figure 1:**
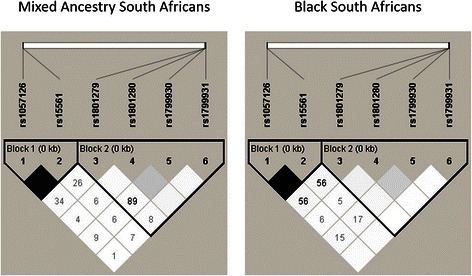
**Linkage disequilibrium between** ***NAT1*** **and** ***NAT2*** **SNPs in Black and Mixed Ancestry South Africans.** Plots show *r*^*2*^ and *D’* values for pairwise LD between *NAT1* and *NAT2* variants in the South African Black and Mixed Ancestry population groups. The left triangle in each plot represents *NAT1* SNPs, while the right triangle represents *NAT2* SNPs. Colour intensity of squares (black to white) indicates the strength of LD (high to low) by *D’*, while numbers within squares refer to *r*^*2*^ values for pairwise SNPs. *D’* and *r*^*2*^ refer to different statistical methods to measure linkage disequilibrium between alleles; *r*^*2*^ is preferred to predict one allele given the other, whereas *D’* is mainly used to assess recombination patterns such as haplotype blocks. Linkage disequilibrium analysis was carried out in control groups by using Haploview [34].

The results of the linkage disequilibrium analysis (LD) and Hardy-Weinberg equilibrium tests in the two South African populations showed that the distribution of genotypes for each SNP in the control groups were in Hardy-Weinberg equilibrium (*P* > 0.05; see Additional file 2). The *NAT1* 1088T>A and 1095C>A SNPs occurred at high frequency (MAF ≥ 0.401) and were in complete LD in both ethnic groups (*D*^*I*^ = 1.0). For the *NAT2* SNPs, only 341T>C (NAT2*5) and 590G>A (NAT2*6) were present at relatively high frequencies (MAF ≥ 0.214) and strong LD (*D*^*I*^ = 1.0) existed between these two polymorphisms in both population groups. Linkage disequilibrium of 857G>A (NAT2*7) and 191G>A (NAT2*14) with other SNPs was not considered because of the low frequency of these two polymorphic variants in both population groups (MAF ≤ 0.063). No significant LD was observed between SNPs in the *NAT1* locus and the two most common SNPs in the *NAT2* locus (341T>C and 590G>A) in either ethnic group, thereby forming two main LD blocks, Block 1 (including *NAT1* SNPs) and Block 2 (including *NAT2* SNPs), as shown in Figure 1.

### NAT1 and NAT2 polymorphisms, environmental exposure, and OSCC risk

A significant association was detected among carriers of the 341 CC variant genotype (NAT2*5/*5) compared to carriers of the wild-type (341 TT) genotype (OR = 0.31; 95% CI 0.11-0.87; *P* = 0.026) in the Mixed Ancestry population, with a borderline association in the Black population (OR = 0.55; 95% CI 0.30-0.99; *P* = 0.05) (Table 2). The *NAT2* 341 C allele (NAT2*5) was associated with a reduced risk for OSCC in the Mixed Ancestry group (OR = 0.57; 95% CI 0.38-0.87; *P* = 0.010), while no significant association was found in the Black group (OR = 0.82; 95% CI 0.64-1.06; *P* = 0.132; see Additional file 3). However, none of the associations observed in the Mixed Ancestry population had significance below the Bonferroni corrected *P*-value of 0.0042.

**Table 2:** **Genotype frequency of** ***NAT2*** **and** ***NAT1*** **polymorphisms and association with OSCC in Black and Mixed Ancestry South Africans**

			Black				Mixed Ancestry			
Gene	SNP	Polymorphism	Controls (%)	Cases (%)	OR (95% CI)^a^	*P*-value	Controls (%)	Cases (%)	OR (95% CI)^a^	*P*-value
*NAT2*	590G>A	G / G	296 (62.3)	257 (57.2)	1 (Ref)	-	172 (59.7)	159 (60.0)	1 (Ref)	-
	(NAT2*6)	G / A	155 (32.6)	163 (36.3)	1.20 (0.85 - 1.69)	0.299	105 (36.4)	93 (35.1)	0.69 (0.39 - 1.21)	0.192
	rs1799930	A / A	24 (5.0)	29 (6.4)	1.46 (0.72 - 2.94)	0.291	11 (3.8)	13 (4.9)	1.30 (0.34 - 4.91)	0.697
	341T>C	T / T	247 (52.1)	237 (53.0)	1 (Ref)	-	130 (45.1)	147 (55.7)	1 (Ref)	-
	(NAT2*5)	T / C	179 (37.8)	178 (39.8)	0.97 (0.69 - 1.36)	0.856	125 (43.4)	101 (38.2)	0.59 (0.34 - 1.04)	0.069
	rs1801280	C / C	48 (10.1)	32 (7.1)	0.55 (0.30 - 0.99)	0.05	33 (11.5)	16 (6.1)	0.31 (0.11 - 0.87)	0.026
	857G>A	G / G	471 (98.9)	440 (98.2)	1 (Ref)	-	265 (92.0)	237 (90.5)	1 (Ref)	-
	(NAT2*7)	G / A	5 (1.0)	7 (1.6)	0.95 (0.19 - 4.77)	0.948	23 (8.0)	24 (9.2)	1.32 (0.54 - 3.23)	0.548
	rs1799931	A / A	0 (0)	1 (0.2)	−		0 (0)	1 (0.4)	−	
	191G>A	G / G	417 (87.6)	402 (90.1)	1 (Ref)	-	275 (95.5)	244 (92.8)	1 (Ref)	-
	(NAT2*14)	G / A	58 (12.2)	41 (9.2)	0.78 (0.46 - 1.33)	0.364	13 (4.5)	18 (6.8)	1.46 (0.48 - 4.49)	0.508
	rs1801279	A / A	1 (0.2)	3 (0.7)	−		0 (0)	1 (0.4)	−	
*NAT1*	1088T>A	T / T	89 (19.2)	100 (22.8)	1 (Ref)	-	106 (37.3)	81 (31.4)	1 (Ref)	-
	(NAT1*10)	T / A	219 (47.3)	196 (44.7)	0.77 (0.50 - 1.18)	0.226	128 (45.1)	130 (50.4)	1.64 (0.90 - 2.98)	0.103
	rs1057126	A / A	155 (33.5)	142 (32.4)	1.07 (0.67 - 1.70)	0.781	50 (17.6)	47 (18.2)	1.57 (0.73 - 3.40)	0.249
	1095C>A	C / C	86 (18.6)	95 (21.7)	1 (Ref)	-	97 (34.1)	69 (26.7)	1 (Ref)	-
	(NAT1*10, NAT1*3)	C / A	220 (47.5)	198 (45.2)	0.79 (0.51 - 1.23)	0.299	130 (45.8)	138 (53.5)	1.83 (0.99 - 3.39)	0.054
	rs15561	A / A	157 (33.9)	145 (33.1)	1.10 (0.69 - 1.75)	0.681	57 (20.1)	51 (19.8)	1.78 (0.82 - 3.84)	0.143

Samples with unknown genotype are not shown.

^a^Odds ratio was adjusted for age, gender, tobacco smoking, and alcohol consumption status; odds ratio was not calculated for subgroups with low sample size.

*Indicates the allele for each of the genes.

The distribution of *NAT2* acetylator phenotypes (rapid, intermediate, and slow acetylators) between cases and control individuals was significantly different in the Mixed Ancestry group, while no significant difference was observed in the Black group, even when intermediate and slow acetylators were merged into one group and compared with the rapid acetylators (Table 3). For the Mixed Ancestry individuals, the *NAT2* slow/intermediate acetylators were at reduced risk of OSCC compared with rapid acetylators (OR = 0.44; 95% CI 0.27-0.70; *P* = 0.001), while the *NAT1* slow/intermediate acetylator status was not a risk factor for OSCC (OR = 0.85; 95% CI 0.43-1.68; *P* = 0.638). For the Black individuals, the ORs were 0.78 (95% CI 0.55-1.11; *P* = 0.166) for *NAT1* slow/intermediate acetylators and 0.82 (95% CI 0.54-1.23; *P* = 0.333) for *NAT2* slow/intermediate acetylators compared to their respective rapid acetylator groups.

**Table 3:** **Distribution of** ***NAT1*** **and** ***NAT2*** **acetylation polymorphisms and association with OSCC in the Black and Mixed Ancestry populations**

		Black				Mixed Ancestry			
Gene	Acetylator status	Controls (%)	Cases (%)	OR (95% CI)^a^	*P*-value	Controls (%)	Cases (%)	OR (95% CI)^a^	*P*-value
*NAT2*	Rapid	87 (18)	90 (19)	1 (Ref)		49 (17)	57 (21)	1 (Ref)	
	Intermediate	231 (48)	192 (41)	0.77 (0.50-1.20)	0.248	124 (43)	113 (42)	0.47 (0.28-0.80)	0.004
	Slow	155 (32)	161 (35)	0.88 (0.56-1.39)	0.588	115 (40)	91 (34)	0.40 (0.24-0.68)	0.001
	Unknown	7 (1)	20 (4)			0 (0)	8 (3)		
	Slow + intermediate	386 (82)	353 (79)	0.82 (0.54-1.23)	0.333	239 (83)	204 (78)	0.44 (0.27-0.70)	0.001
*NAT1*	Rapid	155 (32)	142 (31)	1 (Ref)		50 (17)	47 (17)	1 (Ref)	
	Intermediate	219 (46)	196 (42)	0.72 (0.49-1.05)	0.084	128 (44)	130 (48)	1.04 (0.50-2.17)	0.908
	Slow	89 (18)	100 (22)	0.94 (0.59-1.48)	0.781	106 (37)	81 (30)	0.64 (0.29-1.37)	0.249
	Unknown	17 (3)	25 (5)			4 (1)	11 (4)		
	Slow + intermediate	308 (66)	296 (68)	0.78 (0.55-1.11)	0.166	234 (82)	211 (81)	0.85 (0.43-1.68)	0.638

^a^Odds ratio was adjusted for age, gender, tobacco smoking, and alcohol consumption status.

Ref = reference allele.

*NAT2* rapid = NAT2*4/*4.

*NAT2* intermediate = NAT2*4/*5, NAT2*4/*6, NAT2*4/*7, or NAT2*4/*14.

*NAT2* slow = any combination of NAT2*5, NAT2*6, NAT2*7, and NAT2*14.

*NAT1* rapid = NAT1*10/*10.

*NAT1* intermediate = NAT1*10/*4 or NAT1*10/*3.

*NAT1* slow = NAT1*4/*4, NAT1*4/*3, or NAT1*3/*3.

*Indicates the allele for each of the genes.

The effect of *NAT1* and *NAT2* acetylator phenotypes on the association of tobacco smoking and alcohol consumption with OSCC risk was investigated by case-control analysis (Tables 4 and 5).

**Table 4:** **Association of** ***NAT2*** **acetylator phenotype with OSCC risk upon exposure to environmental risk factors in Black and Mixed Ancestry South Africans**

			*NAT2* rapid acetylators				*NAT2* slow/intermediate acetylators			
Population	Risk factors	Variables	Controls	Cases	OR (95% CI)^a^	*P-*value	Controls	Cases	OR (95% CI)^a^	*P-*value
Black										
	Tobacco smoking	Never smokers	49	39	1 (Ref)		206	132	1 (Ref)	
		Smokers^b^	38	51	2.58 (0.80-8.25)	0.111	180	220	2.76 (1.69-4.52)	<0.0001
	Tot cigarettes/day	0	49	39	1 (Ref)		206	132	1 (Ref)	
		≤9	23	31	2.23 (0.67-7.36)	0.189	112	143	3.27 (1.93-5.54)	<0.0001
		≥10	13	19	3.79 (0.92-15.7)	0.066	58	72	2.49 (1.34-4.62)	0.004
	Alcohol drinking	Non-drinkers	34	39	1 (Ref)		164	127	1 (Ref)	
		Drinkers^c^	53	51	0.94 (0.34-2.62)	0.903	221	224	0.91 (0.59-1.41)	0.675
	Red meat intake^d^	3 times/week or less	−	55			−	198	1 (Ref)	
		Daily or almost daily	−	5			−	31	1.67 (0.61-4.58)	0.316
	White meat intake^d^	3 times/week or less	−	33			−	113	1 (Ref)	
		Daily or almost daily	−	27			−	117	1.28 (0.72-2.29)	0.397
Mixed Ancestry										
	Tobacco smoking	Never smokers	8	2	1 (Ref)		54	12	1 (Ref)	
		Smokers^b^	41	53	−	−	185	191	−	−
	Tot cigarettes/day	0	8	2	1 (Ref)		54	12	1 (Ref)	
		≤9	22	25	−	−	96	58	−	−
		≥10	16	27	−	−	74	129	−	−
	Alcohol drinking	Non-drinkers	15	9	1 (Ref)		100	41	1 (Ref)	
		Drinkers^c^	34	47	2.43 (0.65-9.15)	0.188	138	162	2.77 (1.38-5.58)	0.004
	Red meat intake^d^	3 times/week or less	−	31			−	75	1 (Ref)	
		Daily or almost daily	−	5			−	43	3.55 (1.29-9.82)	0.019
	White meat intake^d^	3 times/week or less	−	20			−	51	1 (Ref)	
		Daily or almost daily	−	16			−	66	1.80 (0.81-4.02)	0.149

Samples with unknown acetylator status are not shown.

Ref = reference allele.

^a^OR was adjusted for age, gender, smoking and alcohol consumption status; OR was not calculated for subgroups with low sample size.

^b^Smokers = current and former smokers.

^c^Drinkers = light to heavy alcohol drinkers.

^d^Case-only analysis has been performed for red meat intake due to lack of data from control individuals.

**Table 5:** **Association of** ***NAT1*** **acetylator phenotype with OSCC risk upon exposure to environmental risk factors in Black and Mixed Ancestry South Africans**

			*NAT1* rapid acetylators				*NAT1* slow/intermediate acetylators			
Population	Risk factors	Variables	Controls	Cases	OR (95% CI)^a^	*P-*value	Controls	Cases	OR (95% CI)^a^	*P-*value
Black										
	Tobacco smoking	Never smokers	84	61	1 (Ref)		166	109	1 (Ref)	
		Smokers^b^	71	81	1.71 (0.73-4.04)	0.218	142	186	3.41 (1.95-5.96)	<0.0001
	Tot cigarettes/day	0	84	61	1 (Ref)		166	109	1 (Ref)	
		≤9	45	50	1.80 (0.72-4.53)	0.208	87	123	3.96 (2.19-7.13)	<0.0001
		≥10	24	31	2.06 (0.73-5.84)	0.174	45	57	2.97 (1.47-5.99)	0.002
	Alcohol drinking	Non-drinkers	66	53	1 (Ref)		130	112	1 (Ref)	
		Drinkers^c^	89	89	1.17 (0.52-2.59)	0.707	177	182	0.87 (0.54-1.40)	0.570
Red meat intake ^d^	3 times/week or less	−	81			−	168	1 (Ref)		
		Daily or almost daily	−	12			−	23	0.93 (0.43-2.0)	0.851
	White meat intake^d^	3 times/week or less	−	56			−	87	1 (Ref)	
		Daily or almost daily	−	38			−	104	1.82 (1.09-3.04)	0.023
Mixed Ancestry										
	Tobacco smoking	Never smokers	17	1	1 (Ref)		43	14	1 (Ref)	
		Smokers^b^	33	46	−	−	191	194	−	−
	Tot cigarettes/day	0	17	1	1 (Ref)		43	14	1 (Ref)	
		≤9	17	24	−	−	101	58	−	−
		≥10	11	20	−	−	77	133	−	−
	Alcohol drinking	Non-drinkers	21	12	1 (Ref)		93	39	1 (Ref)	
		Drinkers^c^	29	35	0.80 (0.19-3.44)	0.764	140	170	3.41 (1.70-6.81)	0.001
	Red meat intake^d^	3 times/week or less	−	22			−	81	1 (Ref)	
		Daily or almost daily	−	7			−	42	1.32 (0.49-3.55)	0.586
	White meat intake^d^	3 times/week or less	−	16			−	56	1 (Ref)	
		Daily or almost daily	−	13			−	66	1.58 (0.68-3.67)	0.288

Samples with unknown acetylator status are not shown.

Ref = reference allele.

^a^OR was adjusted for age, gender, smoking and alcohol consumption status; OR was not calculated for subgroups with low sample size.

^b^Smokers = current and former smokers.

^c^Drinkers = light to heavy alcohol drinkers.

^d^Case-only analysis has been performed for red meat intake due to lack of data from control individuals.

In the Black group, smoking was associated with a significantly increased risk of OSCC for the *NAT2* slow/intermediate acetylators, with an OR of 2.76 (95% CI 1.69-4.52; *P* < 0.0001) among smokers compared to never smokers, while tobacco smoking was not a risk factor for the *NAT2* rapid acetylators (OR = 2.58; 95% CI 0.80-8.25; *P* = 0.111; Table 4). Similarly, the smoking-associated risk for OSCC was significantly higher for *NAT1* slow/intermediate acetylators (OR = 3.41; 95% CI 1.95-5.96; *P* < 0.0001), whereas no significant association was observed for *NAT1* rapid acetylators (OR = 1.71; 95% CI 0.73-4.04; *P* = 0.218; Table 5). However, heterogeneity testing for *NAT2* slow/intermediate acetylators vs rapid acetylators (*I*^*2*^ = 0%; *P* = 0.917) and for *NAT1* slow/intermediate acetylators vs rapid acetylators (*I*^*2*^ = 42.9%; *P* = 0.185) indicated that smoking might increase the risk for OSCC in Black subjects regardless of their acetylator status. The effect of *NAT1* and *NAT2* polymorphisms on the smoking-associated risk of OSCC was not evaluated in the Mixed Ancestry group because of the extremely low number of non-smokers in this group.

The alcohol-related risk of OSCC in the Mixed Ancestry population was significantly increased for *NAT1* slow/intermediate acetylators (OR = 3.41; 95% CI 1.70-6.81; *P* = 0.001) and *NAT2* slow/intermediate acetylators (OR = 2.77; 95% CI 1.38-5.58; *P* = 0.004), whereas no increased risk was observed for alcohol drinkers who were either *NAT1* rapid acetylators (OR = 0.80; 95% CI 0.19-3.44; *P* = 0.764) or *NAT2* rapid acetylators (OR = 2.43; 95% CI 0.65-9.15; *P* = 0.188). However, heterogeneity testing for *NAT2* slow/intermediate acetylators vs rapid acetylators (*I*^*2*^ = 0%; *P* = 0.864) suggested that alcohol may be a significant risk factor for OSCC in both *NAT2* acetylator groups, while substantial heterogeneity between the *NAT1* acetylator phenotypes (*I*^*2*^ = 68.1%; *P* = 0.077) suggested that *NAT1* acetylator status may modify the alcohol-associated risk of OSCC among Mixed Ancestry individuals. In the Black population, the acetylation polymorphism had no effect on the association between alcohol intake and OSCC risk (Tables 4 and 5).

A case-only analysis was performed to investigate the interaction of *NAT1* and *NAT2* acetylator phenotypes with meat consumption on susceptibility to OSCC. In the Mixed Ancestry patients, daily intake of red meat was significantly more frequent among *NAT2* slow/intermediate acetylators than among *NAT2* rapid acetylators (OR = 3.55; 95% CI 1.29-9.82; *P* = 0.019), whereas no significant difference was observed among Black patients (OR = 1.67; 95% CI 0.61-4.58; *P* = 0.316; Table 4). No interaction between *NAT1* acetylator phenotypes and red meat consumption was observed among either Black patients (OR = 0.93; 95% CI 0.43-2.0; *P* = 0.851) or Mixed Ancestry patients (OR = 1.32; 95% CI 0.49-3.55; *P* = 0.586). We also observed a statistically significant interaction between daily consumption of white meat and the *NAT1* slow acetylator status in Black patients (OR = 1.82; 95% CI 1.09-3.04; *P* = 0.023), while such interaction was not significant in Mixed Ancestry patients (OR = 1.58; 95% CI 0.68-3.67; *P* = 0.288; Table 5).

## Discussion

We determined the distribution of the NAT1*3, NAT1*4, NAT1*10, NAT2*4, NAT2*5, NAT2*6, NAT2*7, and NAT2*14 alleles in the Black and Mixed Ancestry populations of South Africa, accounting for the majority of the *NAT1* and *NAT2* alleles observed in Black South Africans and Caucasians [19].

The overall frequency of the *NAT2* slow acetylator alleles in Black Xhosa and Mixed Ancestry healthy individuals (57.2% and 61.5%, respectively) was similar to that previously reported in the Black Tswana and Mixed Ancestry populations from South Africa (59% and 54%, respectively) [19,36]. The allele frequency of NAT1*10 observed in Black Xhosa (57.1%) was similar to that previously reported in Black Tswana (50.5%), whereas the frequency in Mixed Ancestry individuals (40.1%) was closer to that observed in Black South Africans than that of UK Caucasians (18.7%) [19].

The high LD observed between 341T>C (NAT2*5) and 590G>A (NAT2*6) in both South African populations has also been reported in a Northern Indian population [26]. Although studies reported a strong LD between NAT1*10 and NAT2*4 in Caucasians [37,38], we found no significant LD between *NAT1* and *NAT2* alleles in South Africans.

This study confirmed previous findings on the association between tobacco smoking and a high risk of OSCC in the Black and Mixed Ancestry populations from South Africa, as well as a cumulative risk associated with the number of cigarettes smoked per day in the Mixed Ancestry population [4]. Alcohol consumption was an independent risk factor only in the Mixed Ancestry population, but no association was observed in the Black Xhosa population, which is in agreement with the results from a previous study conducted in the same ethnic groups [4]. We also confirmed the previously reported combined effect of alcohol consumption and tobacco smoking on the enhanced oesophageal cancer risk in Black South Africans [39]. Furthermore, tobacco smoking, alcohol consumption, and frequent red meat intake were more prevalent in the Mixed Ancestry population than in the Black population, indicating that Mixed Ancestry South Africans may be more exposed to environmental carcinogens than Black South Africans.

The *NAT2* 341T>C (NAT2*5) polymorphism and *NAT2* slow/intermediate acetylator status were associated with a significantly reduced risk of OSCC in the Mixed Ancestry population. Because NAT2*5 is a slow acetylator allele and NATs are able to activate arylamine carcinogens to reactive intermediates [11,15], the *NAT2* slow acetylator status may result in an enzyme with impaired N-acetylation capacity, possibly leading to reduced production of reactive intermediates and ultimately preventing DNA damage and carcinogenesis. NAT2*5 is the most frequent *NAT2* allele in the South African population, and has the greatest reduction in acetylation activity among *NAT2* slow acetylator alleles [40]. These findings may explain why the association with OSCC was observed among the NAT2*5 allele carriers and not among subjects carrying other *NAT2* slow acetylator alleles. A recent study in a Chinese population found the CC genotype of the *NAT2* rs1565684 T > C polymorphism to be associated with a borderline significantly increased risk for OSCC (OR = 1.77; 95% CI 0.97–3.21; *P* = 0.063). Although this variant has been reported to be functional and in LD with another important *NAT2* polymorphism (rs4345600; *r*^*2*^ = 0.845) in the Han Chinese, the etiology of *NAT2* rs1565684 on oesophageal carcinogenesis is still unknown [41]. In accordance with a previous study on oesophageal adenocarcinoma [29], we found no statistically significant association between *NAT1* polymorphisms (NAT1*10 and *3) and OSCC risk in both South African populations.

When we evaluated the association between the *NAT1* and *NAT2* acetylator phenotypes and OSCC risk in conjunction with environmental exposure, the risk associated with tobacco smoking and alcohol intake appeared to be higher among slow/intermediate acetylators than among rapid acetylators, although this difference was not significant. The lack of a significant association between smoking and alcohol intake with OSCC among rapid acetylators is probably due to the small sample size of these subgroups and the resulting low statistical power to detect significant associations. The interaction of the *NAT1* and *NAT2* polymorphisms with tobacco smoking or red meat intake is likely to be due to the role of NATs in the metabolism of HCAs and nitrosamines, which are the most relevant carcinogen in tobacco smoke and overcooked meat [9,10]. Our finding of an interaction between *NAT1* acetylator status and white meat intake in the Black population is in agreement with a study showing that heat-treated poultry products have significant amounts of HCAs based on the cooking methods and conditions [42]. Furthermore, a previous cohort study reported a significant positive association between poultry intake and oesophageal adenocarcinoma risk [43]. Slow and intermediate acetylators may be unable to detoxify carcinogenic metabolites efficiently; therefore, an impaired detoxification of HCA- and nitrosamine-derived metabolites may explain the greater risk of OSCC associated with low N-acetylation activity upon exposure to environmental carcinogens. However, no study to date has biologically demonstrated that low N-acetylation activity leads to an impaired deactivation of arylamine carcinogens in humans.

A recent cohort study identified statistically significant positive associations of both heme iron (HR = 1.67; 95% CI 1.05–2.68; *P* = 0.022) and processed red meat intake (HR = 2.27; 95% CI 1.33–3.89; *P* = 0.004) with oesophageal adenocarcinoma [44]. Heme iron is an organic form of iron and is mainly provided by red meat. It has been demonstrated that heme iron specifically contributes to carcinogenesis by increasing oxidative stress or by catalysing endogenous formation of nitroso-compounds [45,46]. Thus, the N-nitrosation induced by heme iron may increase the level of nitrosamines in humans and ultimately contribute to oesophageal carcinogenesis in slow/intermediate acetylators [44].

We observed that *NAT1* and *NAT2* acetylator phenotypes modify the alcohol-associated risk of OSCC in the Mixed Ancestry population. Ethanol from alcoholic beverages can cause local irritation of the upper gastrointestinal tract and can act as solvent for tobacco-related carcinogens, facilitating their uptake through the oesophageal mucosa [47]. Furthermore, studies *in vitro* and *in vivo* reported that acetaldehyde, a metabolite of ethanol oxidation, has direct mutagenic and carcinogenic properties on human tissues [48]. A previous study did not find a modulating effect of *NAT2* polymorphisms on the association between alcohol consumption and OSCC risk [26]; however, another study suggested a direct role of *NAT2* in the metabolism of alcohol-derived carcinogens [49]. Impairment of aldehyde dehydrogenase 2 (*ALDH2*), a major enzyme involved in alcohol metabolism, has been associated with acetaldehyde-derived DNA damage in the oesophagus following alcohol ingestion [50]. It is therefore possible that the alcohol-associated risk of OSCC observed among slow/intermediate acetylators in the Mixed Ancestry population may occur by a similar mechanism.

This study assessed the role of *NAT1* polymorphisms on susceptibility to OSCC, the interaction of NATs with alcohol and meat consumption on OSCC risk, and the role of NATs on cancer susceptibility in African populations. A limitation of the study was the lack of data for control individuals with regard to red and white meat intake and the cooking and preparation methods of the meat. Therefore, some caution must be taken when drawing interpretations of the results on the association between meat consumption and oesophageal cancer risk.

## Conclusions

In conclusion, we provide evidence of tobacco smoking, alcohol consumption, and meat intake as independent risk factors for OSCC in South Africa. The main finding of this study is that *NAT1* and *NAT2* polymorphisms may modulate the risk of OSCC upon exposure to environmental risk factors in two indigenous populations of South Africa, indicating that NATs play a key role in detoxification of carcinogens responsible for the initiation of oesophageal cancer. Our findings may offer opportunities to adopt prevention strategies in lifestyle and dietary habits aimed at reducing the exposure to environmental risk factors and at preventing the occurrence of oesophageal cancer in high-risk populations of South Africa.

## Additional files

Additional file 1:
**Primers and fluorogenic probes for** ***NAT1*10*** **and** ***NAT1*3*** **allele determination by TaqMan assay.**

Additional file 2:
**Linkage disequilibrium analysis for** ***NAT1*** **and** ***NAT2*** **SNPs in Black and Mixed Ancestry South Africans.**

Additional file 3:
**Allele frequency of** ***NAT2*** **and** ***NAT1*** **polymorphisms and association with OSCC in Black and Mixed Ancestry South Africans.**

## Abbreviations

NATs: N-acetyltransferases
OSCC: Oesophageal squamous cell carcinoma
HCAs: Heterocyclic amines
SNPs: Single nucleotide polymorphisms
LD: Linkage disequilibrium
