# Erratum to: *NAT1* and *NAT2* genetic polymorphisms and environmental exposure as risk factors for oesophageal squamous cell carcinoma: a case-control study

**DOI:** 10.1186/s12885-015-1681-3

**Published:** 2015-10-07

**Authors:** Marco Matejcic, Matjaz Vogelsang, Yabing Wang, M Iqbal Parker

**Affiliations:** 1 International Centre for Genetic Engineering and Biotechnology, Cape Town Component, Observatory, UCT Medical Campus, Anzio Road, Observatory 7925, Cape Town, South Africa; 2 Division of Medical Biochemistry and IDM, UCT Faculty of Health Sciences, Cape Town, South Africa

Unfortunately, the original version of this article [1] contained an error. The name of the author M. Iqbal Parker was incorrectly spelt as Iqbal M. Parker. The correct spelling is M. Iqbal Parker.
