# Supplementary material for: NAT1 and NAT2 genetic polymorphisms and environmental exposure as risk factors for oesophageal squamous cell carcinoma: a case-control study
Source: BMC Cancer. 2015 Mar 18;15:150. doi: 10.1186/s12885-015-1105-4 (PMC4379954; doi:10.1186/s12885-015-1105-4)
Supplement: Additional file 1: — Primers and fluorogenic probes for NAT1*10 and NAT1*3 allele determination by TaqMan assay. [file 12885_2015_1105_MOESM1_ESM.docx]

**Additional file 1 - Primers and fluorogenic probes for *NAT1*10* and *NAT1*3* allele determination by TaqMan assay**

| **Primer or probe code** | **Sequence** |
| --- | --- |
| 1095/1088-Forward primer | 5’-CACCTATAAAAATGTCATCATA-3’ |
| 1095/1088-Reverse primer | 5’-TCACCAATTTCCAAGATA-3’ |
| 1095C/1088T-LNA-modified probe | FAM-TAAAA**G**ACATTT**A**TTATTATTATTATTA-Q |
| 1095A/1088A-LNA-modified probe | HEX-TAAAA**T**ACATTT**T**TTATTATTATTATTA-Q |
| 1095A/1088T-LNA-modified probe | Cy5-TAAAA**T**ACATTT**A**TTATTATTATTATTA-Q |
| Nucleotides in bold refer to 1095C>A and 1088T>A substitutions  The primers and hybridization probes were designed by Roche Diagnostics Inc. | |
