# Supplementary material for: NAT1 and NAT2 genetic polymorphisms and environmental exposure as risk factors for oesophageal squamous cell carcinoma: a case-control study
Source: BMC Cancer. 2015 Mar 18;15:150. doi: 10.1186/s12885-015-1105-4 (PMC4379954; doi:10.1186/s12885-015-1105-4)
Supplement: Additional file 2: — Linkage disequilibrium analysis for NAT1 and NAT2 SNPs in Black and Mixed Ancestry South Africans. [file 12885_2015_1105_MOESM2_ESM.docx]

**Additional file 2 - Linkage disequilibrium analysis for *NAT1* and *NAT2* SNPs in Black and Mixed Ancestry South Africans**

| **Black** |  |  |  |  |  |  | **Pairwise linkage equilibrium coefficient (*D'*, *r^2^*)** ^c^ | | | | | | | | | |
| --- | --- | --- | --- | --- | --- | --- | --- | --- | --- | --- | --- | --- | --- | --- | --- | --- |
| **Gene** | **SNP** | **rs number** | **Variant** | **Allele** | ***P*-value**  ^a^ | **MAF** ^b^ | *NAT1* | |  | | *NAT2* | |  | |  |  |
|  |  |  |  |  |  |  | 1088T>A | | 1095C>A | | 191G>A | | 341T>C | | 590G>A | 857G>A |
| *NAT1* | 1088T>A | rs1057126 | A | *NAT1*10* | 0.53 | 0.571 | ‒ | | 1 | | 0.56 | | 0.07 | | 0.16 | 0.96 |
|  | 1095C>A | rs15561 | A | *NAT1*10, NAT1*3* | 0.64 | 0.577 | 0.98 | | ‒ | | 0.57 | | 0.05 | | 0.17 | 0.96 |
| *NAT2* | 191G>A | rs1801279 | A | *NAT1*14* | 0.86 | 0.063 | 0.02 | | 0.02 | | ‒ | | 1 | | 1 | 1 |
|  | 341T>C | rs1801280 | C | *NAT1*5* | 0.09 | 0.290 | 0 | | 0 | | 0.03 | | ‒ | | 1 | 1 |
|  | 590G>A | rs1799930 | A | *NAT1*6* | 0.66 | 0.214 | 0.01 | | 0.01 | | 0.02 | | 0.11 | | ‒ | 1 |
|  | 857G>A | rs1799931 | A | *NAT1*7* | 1 | 0.005 | 0.01 | | 0.01 | | 0 | | 0 | | 0 | ‒ |
|  |  |  |  |  |  |  |  | |  | |  | |  | |  |  |
| **Mixed Ancestry** | | |  |  |  |  | **Pairwise linkage equilibrium coefficient (*D'*, *r^2^*)** ^c^ | | | | | | | | | |
| **Gene** | **SNP** | **rs number** | **Variant** | **Allele** | ***P*-value**  ^a^ | **MAF** ^b^ | *NAT1* |  | | *NAT2* | |  | |  | |  |
|  |  |  |  |  |  |  | 1088T>A | 1095C>A | | 191G>A | | 341T>C | | 590G>A | | 857G>A |
| *NAT1* | 1088T>A | rs1057126 | A | *NAT1*10* | 0.34 | 0.401 | ‒ | 1 | | 0.35 | | 0.04 | | 0.09 | | 0.02 |
|  | 1095C>A | rs15561 | A | *NAT1*10, NAT1*3* | 0.31 | 0.430 | 0.89 | ‒ | | 0.26 | | 0.06 | | 0.06 | | 0.07 |
| *NAT2* | 191G>A | rs1801279 | A | *NAT1*14* | 1 | 0.023 | 0 | 0 | | ‒ | | 1 | | 0.8 | | 0.08 |
|  | 341T>C | rs1801280 | C | *NAT1*5* | 0.83 | 0.332 | 0 | 0 | | 0.01 | | ‒ | | 1 | | 1 |
|  | 590G>A | rs1799930 | A | *NAT1*6* | 0.41 | 0.220 | 0 | 0 | | 0 | | 0.14 | | ‒ | | 0.97 |
|  | 857G>A | rs1799931 | A | *NAT1*7* | 1 | 0.040 | 0 | 0 | | 0 | | 0.02 | | 0.01 | | ‒ |
| Linkage disequilibrium of 857G>A and 191G>A with other SNPs was not considered because of the low frequency of these polymorphic variants in both population groups  ^a^ *P*-value for Hardy Weinberg equilibrium  ^b^ MAF = minor allele frequency  ^c^ *D'* above diagonal; *r^2^* below diagonal | | | | | | | | | | | | | | | | |
