# Supplementary material for: NAT1 and NAT2 genetic polymorphisms and environmental exposure as risk factors for oesophageal squamous cell carcinoma: a case-control study
Source: BMC Cancer. 2015 Mar 18;15:150. doi: 10.1186/s12885-015-1105-4 (PMC4379954; doi:10.1186/s12885-015-1105-4)
Supplement: Additional file 3: — Allele frequency of NAT2 and NAT1 polymorphisms and association with OSCC in Black and Mixed Ancestry South Africans. [file 12885_2015_1105_MOESM3_ESM.docx]

**Additional file 3 - Allele frequency of *NAT2* and *NAT1* polymorphisms and association with OSCC in Black and Mixed Ancestry South Africans**

|  |  |  | **Black** |  |  |  |  | **Mixed Ancestry** | |  |  |
| --- | --- | --- | --- | --- | --- | --- | --- | --- | --- | --- | --- |
| **Gene** | **SNP** | **Polymorphism** | **Control (%)** | **Cases (%)** | **OR (95% CI) ^a^** | ***P*-value** |  | **Control (%)** | **Cases (%)** | **OR (95% CI) ^a^** | ***P*-value** |
| *NAT2* | 590G>A  (NAT2*6)  rs1799930 | G | 747 (78.6) | 676 (75,3) | 1 (Ref) | - |  | 449 (77.9) | 411 (77.5) | 1 (Ref) | - |
|  |  | A | 203 (21.4) | 222 (24.7) | 1.21 (0.92 - 1.59) | 0.166 |  | 127 (22.0) | 119 (22.4) | 0.85 (0.55 - 1.33) | 0.483 |
|  | 341T>C  (NAT2*5)  rs1801280 | T | 673 (70.9) | 652 (72.9) | 1 (Ref) | - |  | 385 (66.8) | 395 (74.8) | 1 (Ref) | - |
|  |  | C | 275 (29.0) | 242 (27.1) | 0.82 (0.64 - 1.06) | 0.132 |  | 191 (33.2) | 133 (25.2) | 0.57 (0.38 - 0.87) | **0.010** |
|  | 857G>A  (NAT2*7)  rs1799931 | G | 947 (99.5) | 887 (98.9) | 1 (Ref) | - |  | 553 (96.0) | 498 (95.0) | 1 (Ref) | - |
|  |  | A | 5 (0.5) | 9 (1.0) | 2.01 (0.17 - 8.70) | 0.348 |  | 23 (4.0) | 26 (5.0) | 1.30 (0.55 – 3.11) | 0.549 |
|  | 191G>A  (NAT2*14)  rs1801279 | G | 892 (93.7) | 845 (94.7) | 1 (Ref) | - |  | 563 (97.7) | 506 (96.2) | 1 (Ref) | - |
|  |  | A | 60 (6.3) | 47 (5.3) | 0.88 (0.54 - 1.45) | 0.612 |  | 13 (2.3) | 20 (3.8) | 1.47 (0.49 - 4.38) | 0.494 |
| *NAT1* | 1088T>A  (NAT1*10)  rs1057126 | T | 397 (42.9) | 396 (45.2) | 1 (Ref) | - |  | 340 (59.8) | 292 (56.6) | 1 (Ref) | - |
|  |  | A | 529 (57.1) | 480 (54.8) | 1.07 (0.85 - 1.35) | 0.578 |  | 228 (40.1) | 224 (43.4) | 1.31 (0.90 - 1.92) | 0.158 |
|  | 1095C>A  (NAT1*10, NAT1*3)  rs15561 | C | 392 (42.3) | 388 (44.3) | 1 (Ref) | - |  | 324 (57.0) | 276 (53.5) | 1 (Ref) | - |
|  |  | A | 534 (57.7) | 488 (55.7) | 1.09 (0.86 - 1.37) | 0.482 |  | 244 (43.0) | 240 (46.5) | 1.37 (0.94 -2.00) | 0.099 |
| ^a^ Odds ratio was adjusted for age, gender, tobacco smoking and alcohol consumption status  Ref = reference allele | | | | | | | |  |  |  |  |
|  | | | | | | | | | | | |
